# A phase I/II pharmacokinetics/pharmacodynamics study of irinotecan combined with S−1 for recurrent/metastatic breast cancer in patients with selected *UGT1A1* genotypes (the JBCRG‐M01 study)

**DOI:** 10.1002/cam4.1258

**Published:** 2017-11-13

**Authors:** Hiroshi Ishiguro, Shigehira Saji, Shogo Nomura, Sunao Tanaka, Takayuki Ueno, Masahide Onoue, Hiroji Iwata, Takeharu Yamanaka, Yasutsuna Sasaki, Masakazu Toi

**Affiliations:** ^1^ Department of Medical Oncology International University of Health and Welfare Hospital Nasushiobara Japan; ^2^ Department of Medical Oncology Fukushima Medical University Fukushima Japan; ^3^ Center for Research and Administration and Support National Cancer Center Kashiwa Japan; ^4^ Department of Breast Surgery Graduate School of Medicine Kyoto University Kyoto Japan; ^5^ Department of Breast Surgery Kyorin University Hospital Mitaka Japan; ^6^ Department of Pharmacy Kitano Hospital Osaka Japan; ^7^ Department of Breast Oncology Aichi Cancer Center Nagoya Japan; ^8^ Department of Biostatistics Yokohama City University Yokohama Japan; ^9^ Department of Medical Oncology Showa University Tokyo Japan

**Keywords:** Breast Cancer, circulating endothelial cells, Irinotecan, S‐1, SN‐38, *UGT1A1*

## Abstract

S‐1 and irinotecan combination is attractive for breast cancer refractory to anthracyclines and taxanes. Patients with advanced human epidermal growth factor receptor 2 (HER2)‐negative breast cancer previously treated with anthracyclines and taxanes were eligible. Patients with brain metastases and homozygous for UGT1A1 *6 or *28 or compound heterozygous (*6/*28) were excluded. A dose‐escalation design was chosen for the phase I portion (level 1: irinotecan 80 mg/m^2^ days 1–8 and S‐1 80 mg/m^2^ days 1–14 every 3 weeks; level 2: irinotecan 100 mg/m^2^ and S‐1 80 mg/m^2^). Study objectives included determination of the recommended dose for phase II, response rate, progression‐free survival (PFS), and safety. Pharmacokinetics and CD34^+^ circulating endothelial cells (CECs) as pharmacodynamics were also analyzed. Thirty‐seven patients were included. One patient at each level developed dose‐limiting toxicities; therefore, level 2 was the recommended dose for phase II. Diarrhea was more common in patients possessing a *6 or *28 allele compared with wild‐type homozygous patients (46% and 25%). Among 29 patients treated at level 2, PFS was longer for UGT1A1 wt/*6 and wt/*28 patients than for *wt*/*wt* patients (12 vs. 8 months, *P* = 0.06). PFS was significantly longer in patients with a larger‐than‐median SN‐38 area under the curve (AUC) than in those with a smaller AUC (*P* = 0.039). There was an association between clinical benefit and reduction in baseline CD34^+^ CECs by S‐1 (*P* = 0.047). The combination of irinotecan and S‐1 is effective and warrants further investigation.

## Introduction

There is an increasing demand for effective regimens for patients with recurrent/metastatic HER2‐negative breast cancer for whom anthracyclines and taxanes have failed. S‐1 is an oral anticancer agent that contains gimeracil, oteracil, and tegafur (a prodrug of 5‐FU) which has been shown to be active against breast cancer 1. Irinotecan exhibits an antitumor effect against recurrent/metastatic breast cancer that has been pretreated with anthracyclines and taxanes 2, 3. Because S‐1 and irinotecan have different mechanisms of action to those of anthracyclines and taxanes, and a synergistic effect of 5‐FU and irinotecan has been noted 4, their combination is attractive. Furthermore, intratumoral dihydropyrimidine dehydrogenases (DPDs) are often elevated in breast cancer, and the incorporation of a DPD inhibitor, as in S‐1, might be advantageous 5.

SN‐38 is an active metabolite of irinotecan, that is, excreted after conjugation to form the inactive compound SN‐38G with glucuronic acid by uridine diphosphate (UDP)‐glucuronosyl transferase (UGT) 1A1. Variants of the gene for *UGT1A1* exist, and in patients with certain polymorphisms (those who are homozygous for UGT1A1*6/*6 or UGT1A1*28/*28, or heterozygous, UGT1A1*6/*28) glucuronidation is impaired, resulting in an increase in the risk of serious adverse reactions to irinotecan 6, 7, 8.

Circulating endothelial cells (CECs) comprise two distinct populations: vascular‐derived mature CECs, elicited by damage to the endothelium of the vasculature, and bone‐marrow‐derived circulating endothelial progenitors (CEPs), which contribute to neovascularization. Although CD34 is expressed not only in endothelial progenitor cells but also in some of the mature endothelial cells, it has been widely used to identify progenitor cells with clonogenic potential 9. A reduction in CEPs is strongly correlated with antiangiogenic effects 10. Because vascular endothelial growth factor‐A‐driven tumor angiogenesis for the formation of a functional vascular bed and the subsequent tumor growth partly depend on the mobilization of CEPs, a change in the CEP level may be a predictive marker for antiangiogenesis therapy 11. In our previous studies 12, 13, the CD34^+^ CEC level was closely associated with the treatment response to chemotherapy, including S‐1.

We therefore conducted a phase I/II study of combined therapy with irinotecan plus S‐1 in patients with recurrent/metastatic breast cancer who had already received both anthracyclines and taxanes, including analyses of pharmacokinetics, pharmacogenomics, and CECs/CEPs as potential pharmacodynamic markers.

## Methods

### Patients

Patients were included if they had pathologically confirmed recurrent and/or metastatic human epidermal growth factor receptor 2 (HER2)‐negative breast cancer, were aged 75 or younger and had previously received anthracycline and taxane chemotherapy (including adjuvant and/or neoadjuvant use). All patients were required to have a measurable lesion according to the Response Evaluation Criteria in Solid Tumors (RECIST) guideline version 1.0 14. Other criteria included an Eastern Cooperative Oncology Group performance status of 0–2, expected survival of longer than 12 weeks, adequate organ function, a white blood cell count of ≥3000/mm^3^ or an absolute neutrophil count of ≥1500/mm^3^, a platelet count of ≥10 × 10^4^/mm^3^, a hemoglobin level of ≥9.0 g/dL, aspartate transaminase (AST)/alanine aminotransferase (ALT) levels of ≤2 × the upper normal limit, total bilirubin ≤1.5 mg/dL, a creatinine level of ≤1.2 mg/dL, estimated creatinine clearance of ≥50 mL/min, and oxygen saturation >90%. All patients provided written informed consent. Patients were excluded if they had brain metastases, diarrhea, ileus, clinically significant infection, a significant amount of fluid retention, interstitial pneumonitis, or another clinically significant condition. Eligible patients were preregistered and a test for *UGT1A1* genetic polymorphism was performed. Patients who were homozygous for UGT1A1*6 or *28, or compound heterozygous (6/*28), were excluded.

### Study design

This was a phase I/II, multicenter clinical trial. Phase I was a dose‐escalation study using the standard 3 + 3 design. Patients received 80 (level 1) or 100 mg/m^2^ (level 2) of intravenous irinotecan on days 1 and 8 plus 80 mg/m^2^/day of oral S‐1 from day 1 through day 14. Cycles were repeated every 21 days until the prespecified discontinuation criteria was met. Dose‐limiting toxicities were defined as ≥grade 3 nonhematological toxicities, grade 4 neutropenia, or febrile neutropenia during the first cycle. The maximum tolerated dose (MTD) was defined as the dose at which more than one patient in every three patients experienced dose‐limiting toxicities. The dose one level lower than the MTD (or level 2 if no MTD was observed) was defined as the recommended dose.

The primary endpoint of phase II was response and clinical benefit rate. Secondary endpoints included safety, progression‐free survival (PFS), and overall survival (OS). The study protocol was approved by the institutional review board at each participating study center before initiation and was conducted in accordance with the Ethical Guideline for Clinical Studies and Human Genome/Gene Analysis Research by the Ministry of Health, Labour and Welfare, Japan and the Declaration of Helsinki. The study was registered in the University Hospital Medical Information Network Clinical Trial Registry managed by the National University Hospital Council of Japan and operated by the University of Tokyo Hospital (UMIN000000517). All patient information forms were collected and managed by the Japan Breast Cancer Research Group (JBCRG) data center.

### Study assessment

Adverse events were graded according to the National Cancer Institute Common Terminology Criteria for Adverse Events, version 3.0. Tumor assessment was conducted every two cycles. Response was assessed based on RECIST version 1.0. Partial response (PR) and complete response (CR) were confirmed by repeated assessment at least 4 weeks apart. Stable disease (SD) was evaluated at least 8 weeks after study treatment began; if it continued beyond 24 weeks, it was defined as long SD. Clinical benefit was defined as either CR, PR, or long SD. PFS was defined as the time between treatment initiation and disease progression or death, whichever occurred first. Response duration was defined as the PFS only of patients whose disease responded. OS was defined as the time between treatment initiation and death due to any cause. PFS and OS were censored at the last day of tumor or survival assessment, respectively.

### Pharmacogenomics, pharmacokinetics, and pharmacodynamics

Pharmacogenomics (PGx) analysis for UGT1A1 *6 and *28 was conducted by Bio Medical Laboratories, Inc. The pharmacokinetics (PK) of irinotecan were assessed during the first cycle for patients enrolled at Kyoto University Hospital, with additional informed consent. Blood samples were obtained prior to irinotecan infusion and at 10, 60, 120, and 240 min after the end of infusion. Plasma concentrations of irinotecan, SN‐38, and SN‐38G were measured by high‐performance liquid chromatography at ADME & Tox. Research Institute, Sekisui Medical Co. Ltd, Ibaraki, Japan. PK parameters were calculated with WinNonlin Professional ver. 5.0.1 software (Pharsight Co., Mountain View, CA, USA) using a linear trapezoidal model. For pharmacodynamics (PD) analysis, we used CD34^+^ CECs as a marker for CEPs. CD34^+^ CECs were measured using a CellSearch system (Veridex LLC, Co. Ltd, NJ, USA). CECs were classified as 4,6‐diamino‐2‐phenylindole (DAPI)^+^, CD45^−^, CD146^+^, or CD105^+^ and were subsequently stained with an anti‐CD34 antibody and evaluated with an extra channel in the CellSearch system.

### Statistical analysis

The main objective of the phase II portion of the study was to test the null hypothesis that the response rate (RR) is 10% against the alternative of 30% using a single‐stage binomial study design. An RR of <10% was deemed unacceptable based on previous data from a study of S‐1 1. At least 33 patients were required in phase II with a one‐sided alpha of 0.05 and a beta of 0.2. The Kaplan–Meier method was used to estimate survival curves, and the log‐rank test and univariate Cox proportional hazards model were used to evaluate the association between *UGT1A1* genetic polymorphisms and survival outcomes. The Mann–Whitney *U*‐test was used to compare CD34^+^ CEC counts during combination treatment. All reported *P* values were two‐sided, *P* *<* 0.05 was considered statistically significant, and 90% confidence intervals (CIs) were calculated with the Clopper and Pearson method. Statistical analyses were performed with SAS Release 9.3 (SAS Institute Inc., Cary, NC, USA) and JMP v9.0.0 software (SAS Institute Inc., NC, USA).

## Results

### Patient population, clinical endpoints, and pharmacogenomics

**Figure 1:**
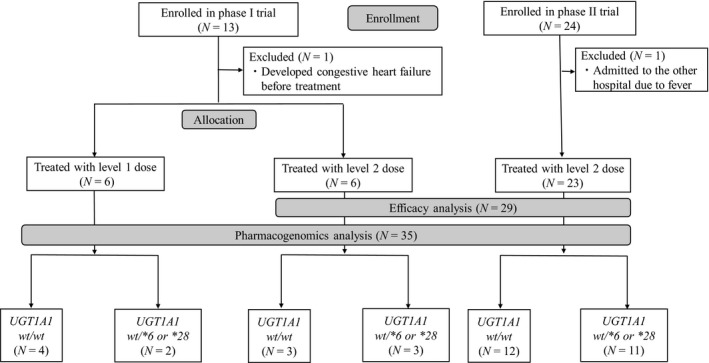
Diagram of clinical trial enrollment and study schema. *wt, wild‐type*.

Thirty‐seven patients were enrolled in this study (13 in phase I and 24 in phase II) between November 2006 and June 2011 (the trial closed before full enrollment due to poor accrual) (Fig. 1). One patient was excluded from each phase, leaving 35 patients for analysis (12 for phase l and 23 for phase II). All patients had received two or more previous chemotherapies other than anthracyclines and taxanes and the majority had visceral metastases such as hepatic disease (Table 1).

**Table 1:** Baseline patient characteristics

Characteristics	Phase I (*N* = 12)	Phase II (*N* = 23)
Age mean (SD)	51.8 (12.4)	55.9 (9.8)
Postmenopausal (%)	6 (50)	17 (74)
Hormone receptor (ER)		
Positive (%)	8 (67)	15 (65)
Negative or unknown (%)	4 (33)	8 (35)
Previous chemotherapy other than anthracycline and taxane		
Two or more (%)	12 (100)	23 (100)
Capecitabine (%)	10 (83)	14 (61)
Vinorelbine (%)	4 (33)	6 (26)
Visceral disease	Not available	19 (83)
Liver (%)	Not available	17 (74)
Lung (%)	Not available	6 (26)

SD, standard deviation; ER, estrogen receptor.

The median number of irinotecan cycles and dose intensity were not different between patients with one of the *UGT1A1* polymorphisms and homozygous wild‐type patients (Table 2).

**Table 2:** Treatment delivery by trial phase

	Phase I				Phase II	
Dose level	Level 1		Level 2		Level 2	
*UGT1A1* genotype	*wt/wt*(*N* = 4)	*wt/mu*(*N* = 2)	*wt/wt*(*N* = 3)	*wt/mu*(*N* = 3)	*wt/wt*(*N* = 12)	*wt/mu*(*N* = 11)
Cycles (median)	10.0	13.5	7.0	8.0	3.0	4.0
Dose intensity (median)(mg/m^2^/week)	26.3	19.7	33.1	33.1	19.5	25.0

**Figure 2:**
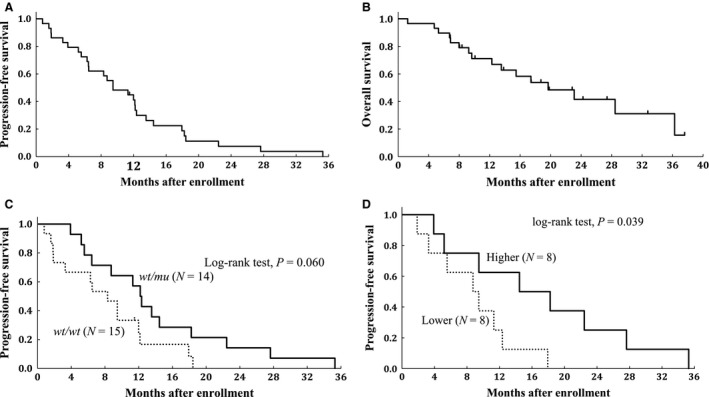
Progression‐free and overall survival of patients at dose level 2. (A, C, D) Progression‐free survival, (B) Overall survival. (A and B) Entire patient population (*N* = 29). (C) By *UGT1A1* genotype (*N* = 29). (D) By SN‐38 area under the curve (*N* = 16). *wt, wild‐type; mu, mutant*; AUC, area under the curve.

Among the 29 patients treated at level 2, RR, clinical benefit rate (CR + PR + long SD), PFS, and OS were numerically better for patients who were heterozygous for *6 or *28 compared with those for patients who were wild‐type homozygous (Table 3). Figures 2A and B show Kaplan–Meyer curves by *UGT1A1* genotype for PFS and OS at treatment level 2. Although not statistically significant, the median PFS (12 vs. 8 months, hazard ratio [HR] 0.47, *P* = 0.060, Fig. 2C) and median OS (23 vs. 17 months, HR 0.74, *P* = 0.56) were longer for the heterozygous patients compared with the homozygous wild‐type patients, respectively.

**Table 3:** Efficacy results by study medication dose level

	Level 1		Level 2		
	Phase I		Phase l & II		
	*wt/wt*(*N* = 4)	*wt/mu*(*N* = 2)	*wt/wt*(*N* = 15)	*wt/mu*(*N* = 14)	Total(*N* = 29)
Best response (%)					
CR	0 (0)	0 (0)	0 (0)	0 (0)	0 (0)
PR	2 (50)	0 (0)	1 (7)	3 (21)	4 (14)
SD	1 (25)	2(100)	7 (47)	6 (43)	13 (45)
PD	0 (0)	0 (0)	1 (7)	0 (0)	1 (4)
Not evaluable	1 (25)	0 (0)	6 (40)	5 (36)	11 (38)
Clinical benefit rate (%)CR + PR + long SD	4 (100)	2 (100)	4 (27)	5 (36)	9 (31)
Median response duration (days) (min–max)	40 (38–41)	NE	350	250 (138–686)	300 (138–686)
Median progression‐free survival (months) (min–max)	12 (6–17)	9 (6–11)	8 (2–12)	12 (6–18)	10 (6–12)
Median overall survival (months) (min–max)	20 (12–25)	NE	17 (7–NE)	23 (8–36)	20 (12–36)

CR, complete response; NE, not estimable; PD, progressive disease; PR, partial response; SD, stable disease.

Diarrhea was more common in patients possessing a *6 or *28 allele compared with wild‐type homozygous patients (Table 4).

**Table 4:** Grade 2 or higher adverse events observed in more than two patients

	Phase 1 (first cycle only)				Phase II (all cycles)	
Dose level	Level 1		Level 2		Level 2	
*UGT1A1* genotype	*wt/wt*(*N* = 4)	*wt/mu*(*N* = 2)	*wt/wt*(*N* = 3)	*wt/mu*(*N* = 3)	*wt/wt*(*N* = 12)	*wt/mu*(*N* = 11)
Diarrhea (%)	1 (25)	0 (0)	0 (0)	0 (0)	3 (25)	5 (46)
Constipation (%)	3 (75)	0 (0)	0 (0)	0 (0)	1 (8)	1 (9)
Vomiting (%)	0 (0)	0 (0)	0 (0)	1 (33)	2 (17)	1 (9)
Anorexia (%)	0 (0)	0 (0)	0 (0)	0 (0)	3 (25)	1 (9)
Fatigue (%)	1 (25)	0 (0)	0 (0)	0 (0)	3 (25)	1 (9)
Febrile neutropenia (%)	0 (0)	0 (0)	0 (0)	0 (0)	2 (17)	0 (0)

*wt*, wild‐type for UGT1A1; *mu*; *6 or *28 for UGT1A1.

### Pharmacokinetics and pharmacodynamics

**Figure 3:**
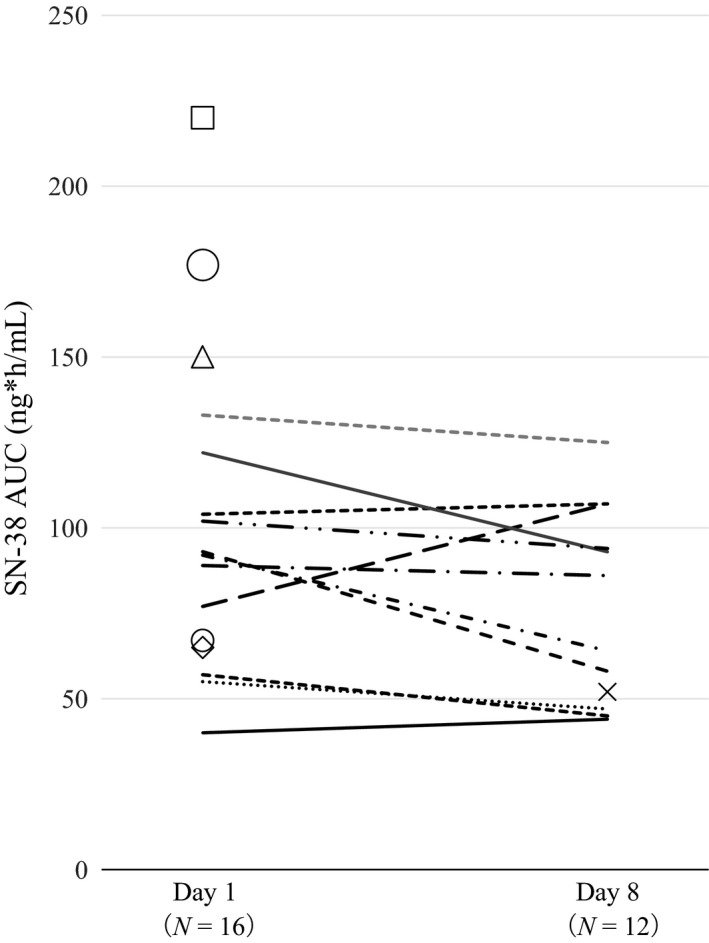
AUCs for SN‐38 on cycle 1 days 1 and 8. Each line connects the SN‐38 AUC of days 1 and 8. Open figures (small and large circles, square, diamond, and triangle) on day 1 indicate that these patients did not receive irinotecan on day 8; therefore, only day 1 AUC results are available.

For 16 patients, the PK of irinotecan, SN‐38 and SN‐38G were assessed during the first cycle on days 1 and 8 (five patients did not receive irinotecan on day 8 due to not meeting administration criteria and one did not provide samples for PK analysis on day 1 due to poor venous access). The level of the active metabolite SN‐38 was constant between day 1 and day 8 of the first treatment cycle and significantly lower in the five patients who could not receive the irinotecan dose on day 8 (Fig. 3). PFS was significantly longer in patients with a larger‐than‐median SN‐38 AUC (*P* = 0.039) than in those with a smaller AUC (Fig. 2D).

**Figure 4:**
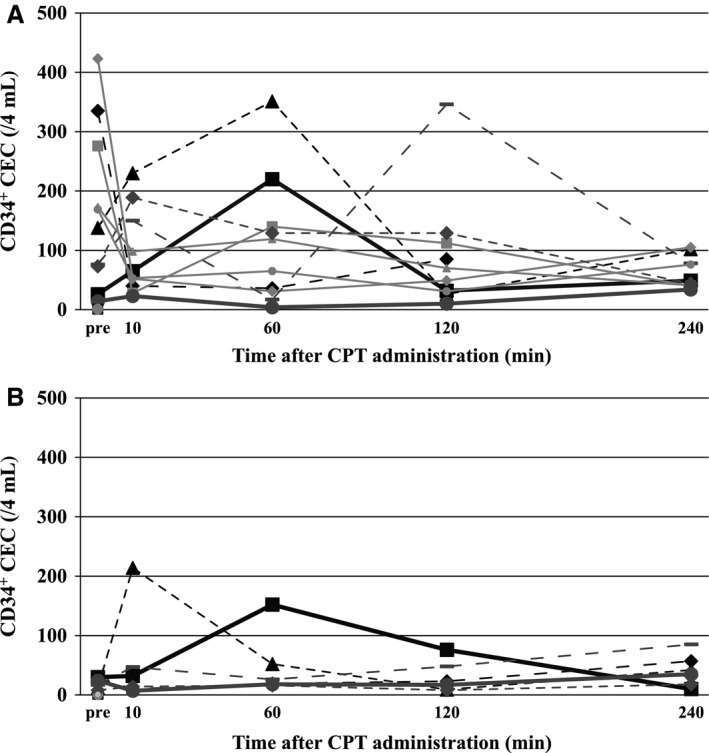
Kinetics of CD34^+^ CECs with irinotecan infusion. (A) day 1 (*N* = 10), (B) day 8 (*N* = 6). Each line corresponds to a single patient and same lines in (A) and (B) correspond to the same patients.

**Figure 5:**
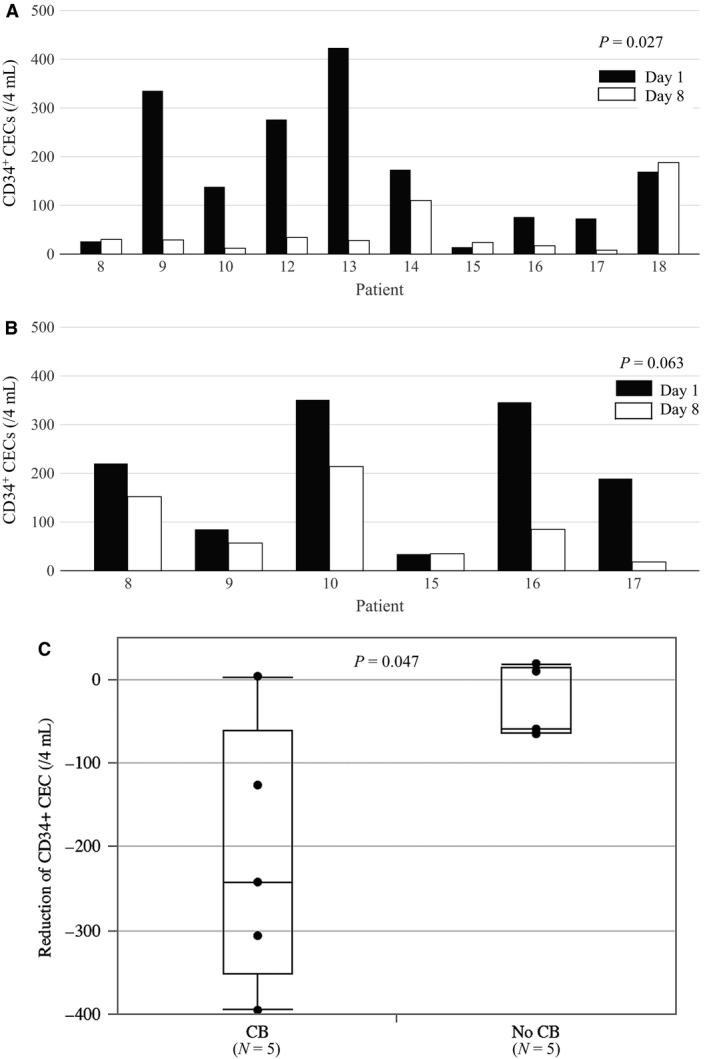
(A) Comparison of the baseline CD34^+^ CEC level before irinotecan administration between days 1 and 8. (B) Comparison of the peak CD34^+^ CEC level after administration of irinotecan between days 1 and 8. (C) Association between reduction in CD34^+^ CEC level and clinical benefit (CB).

Ten patients had CD34^+^ CEC analysis during the first cycle. There was a transient increase in CD34^+^ CEC levels on days 1 and 8 shortly after administering irinotecan (Fig. 4A and B). Baseline levels (just before administering irinotecan) and peak levels (within 4 h of administration) were both lower on day 8 compared with day 1 (Fig. 5A and B, *P* = 0.027, *P* = 0.063, respectively). There was an association between clinical benefit and reduction in baseline CD34^+^ CECs by S‐1 (Fig. 5C, *N* = 10, *P* = 0.047).

## Discussion

This study not only determined the recommended dose and safety/efficacy profile of the combination of S‐1 and irinotecan but also elucidated the PGx, PK, and PD (CD34^+^ CECs). The recommended dose was set at level 2 for all patients with no significant difference in the safety profiles between genetic variants. However, a higher RR and a longer PFS were seen in patients who were heterozygous for a *UGT1A1* mutation compared with homozygous wild‐type patients. This was supported by the PK analysis in which patients with a larger SN‐38 AUC had a significantly longer PFS. In terms of PD, association between clinical benefit and reduction in baseline CD34+ CECs by S‐1 was demonstrated.

This study had several limitations. First, it was not possible to complete enrollment because of slow accrual. Despite the small sample size, however, the RR at level 2 was above the threshold level and associations between the efficacy endpoints and PGx/PK were demonstrated. Second, the PK and PD analyses were conducted in a limited number of patients since the sample collection and analysis was possible only at Kyoto University Hospital. Third, we could not conduct PK analysis of S‐1 because it was not possible to perform such a complex sampling in an outpatient setting. Finally, S‐1 may not be available in several countries, including the United States; therefore, the results may not be generalizable to these countries.

Several studies have shown associations between *UGT1A1* polymorphisms and the toxicity of irinotecan, particularly neutropenia 7, 8, 15. In this study, the combination with S‐1 and a small sample size make analysis difficult. Although diarrhea is another characteristic toxicity of irinotecan, the degree of baseline constipation, a combination with S‐1 and the use of 5HT_3_ antagonist make this analysis difficult with a limited number of patients. A few studies have indicated higher efficacy for patients with *UGT1A1* polymorphisms, with small differences in the safety profiles 16. Our PK analysis supported these polymorphisms being functionally relevant. It is possible that patients without *UGT1A1* polymorphisms may require a higher dose of irinotecan.

For patients with disease refractory to anthracyclines and taxanes, single agent chemotherapy with agents such as irinotecan delivered at the MTD given every few weeks is an option for patients with low tumor burden, but PFS is relatively short and combination chemotherapy strategies are largely not successful 17. We have previously reported that a low baseline CD34^+^ CEC level was associated with higher pathological CR in primary breast cancer 12. Cytotoxic chemotherapy increases the CD34^+^ CEC count, possibly owing to the damage to normal vessels 12. Chemotherapy can be more frequently administered at low doses, resulting in fewer side effects and a better quality of life; this is known as metronomic chemotherapy. Capecitabine and S‐1 meet the characteristics of metronomic chemotherapy. This mode of administration suppresses the induction of endothelial cells from the bone marrow 18 and decreases CD34^+^ CEC levels 13. There could be a good rationale for combining metronomic chemotherapy with intravenous chemotherapy, such as taxanes 19 and irinotecan.

In conclusion, S‐1 and irinotecan combination therapy is well tolerated with a modest RR but a promising clinical benefit rate and PFS. PGx analysis not only helps to avoid significant irinotecan‐related toxicity, as previously reported, but also might identify patients who are more likely to benefit from such a treatment.

## Conflict of Interest

S. Saji, T. Yamanaka, and Y. Sasaki report personal fees from TAIHO PHARMACEUTICAL CO., LTD. H. Ishiguro, S. Saji, and M. Toi report grants from TAIHO PHARMACEUTICAL CO., LTD.

H. Ishiguro, S. Saji, H. Iwata, and M. Toi are board members of JBCRG. The other authors have declared no conflicts of interest.
